# Supplementary material for: DICOM for quantitative imaging biomarker development: a standards based approach to sharing clinical data and structured PET/CT analysis results in head and neck cancer research
Source: PeerJ. 2016 May 24;4:e2057. doi: 10.7717/peerj.2057 (PMC4888317; doi:10.7717/peerj.2057)
Supplement: Appendix S1 [file peerj-04-2057-s001.pdf]

Appendix 1: Background and motivation for using DICOM to encode patient clinical information

In the following, we discuss the motivation for encoding relevant clinical information available for the subjects enrolled in the study, which included clinical history (such as the diagnosis and pathology, surgery and radiotherapy administration, and demographics) and outcomes (follow-up date and status, and the date of death, when applicable).

To the best of our knowledge, there is no consensus in the medical imaging research community on a standard mechanism for sharing the clinical information about the patient cohorts used in quantitative imaging research studies. Most widely used are spreadsheet formats, such as CSV (Comma-Separated Values) or Excel (Microsoft, Redmond, WA). As an example, the Cancer Genome Atlas-Lung Squamous Cell Carcinoma (TCGA-LUSC) data collection is accompanied by an archive of seven space-delimited text files storing information about patient demographics, radiation therapy and drug administration (The Cancer Imaging Archive (TCIA), 2015). Although this data is linked to the imaging data of the collection via common patient identifiers, it cannot be queried in a manner linked to querying of the imaging data, there are no tools to validate those text files, and the lack of a common standard or template used to encode them can lead to difficulties integrating clinical data from different sources.

On the clinical side, various standards exist for communicating clinical data. These include Clinical Document Architecture (CDA®) (Dolin et al., 2006), Health Level Seven (HL7) version 2 messages (Mwogi, Biondich & Grannis, 2014), HL7 version 3 messages (Goossen & Langford, 2014) and Fast Healthcare Interoperability Resources (FHIR) (Bender, Duane & Kamran, 2013; Health Level Seven® International, 2015). A nascent effort is the concept of "Electronic Health Records (EHR) based phenotyping" (Rasmussen et al., 2015), but EHR integration is beyond the scope of this project, if for no other reason an EHR was not the direct integrated source of the clinical information, as opposed to a dedicated research database into which the information had been transcribed.

Given the lack of a consensus about standard mechanism for sharing clinical information, and the expectation that the primary reuse of the image and quantitative result datasets would be in an image-aware context (i.e., within image display and analysis software), it was deemed expedient to develop a template in which to encode the clinical information as DICOM SR. This permits the use of standard codes, hierarchical organization, and reuse of parts of existing templates in the DICOM standard.

The choice of DICOM SR to encode clinical information was motivated by the following factors:

1. *Harmonization.* By using DICOM consistently, we can maintain composite patient context across the objects, enabling consistent linking of the clinical, imaging and quantitative derived information. Recognizing that the source PET/CT data will always be in DICOM, this choice allows a consistent data format across all information types and allows us to focus on only one set of standardized conventions for data encoding.
2. *Interoperability*. The resulting DICOM objects can be stored side-by-side with the images and annotations within readily available DICOM server infrastructure, enabling storage, search, and retrieval using such existing infrastructure deployed for managing medical image data.
3. *Accessibility.* A variety of robust commercial and freely available tools and toolkits exist to interact with DICOM data, which can be applied in uniform manner to both clinical and imaging information. This enables conversion of DICOM representation into other commonly used forms, such as PDF, HTML, XML, JSON, or delimited text. As an example, conversion of DICOM to XML and HTML is supported by DCMTK command line tools, and conversion of XML representation to delimited text can be performed using XSLT. There are also standard mechanisms defined for transcoding some components and/or templates of DICOM SR into HL7 CDA (National Electrical Manufacturers Association (NEMA), 2016).

4. *Validation.* Existing tools can be used to verify the validity of the resulting objects at different levels. Specifically, *dciodvfy* tool (Clunie, 2015a) can be used to check encoding of the DICOM data elements. *com.pixelmed.validate.DicomSRValidator* (Clunie, 2015b) can confirm compliance of the resulting object with a specific SR template, once the template has been added to the tool.

The choice of DICOM SR for this project was motivated not only by the best match of the capabilities of the mechanism to the requirements of the project, but also by the widespread clinical use of SR for similar applications. In those clinical applications for which image-derived results have achieved acceptance, such as echocardiography, obstetric ultrasound, and mammography Computer Aided Detection (CAD) (Brent J. Liu, Anh Le, H.K. Huang, 2015), both manual and automated processes routinely use the DICOM SR mechanism (Dean Bidgood, 1998; Clunie, 2000; Loef & Truyen, 2005) to achieve interoperability. This interoperability transcends modality and Picture Archiving and Communication System (PACS) boundaries to include downstream systems, such as those used for speech recognition driven human reporting systems, which are now also capable of ingesting DICOM SR measurements and codes (i.e., in the PACSgear ModLink (Imaging Technology News, 2013), Nuance PowerScribe 360 (BusinessWire, 2013) and MModal Fluency for Image Reporting (MModal, 2014) products). The recent trend towards the widespread implementation of Radiation Dose SRs (RDSRs) has the potential to greatly extend the breadth of the clinical infrastructure that supports DICOM SR (Talati et al., 2013). Though a less common application for it, DICOM SR can and has been used for encoding human-generated reports (i.e., those dictated by a radiologist) (Noumeir, 2006), and as a consequence is supported in common off-the-shelf image viewing tools, such as OsiriX (Rosset, Spadola & Ratib, 2004). DICOM SR has also been used for more exotic applications such as organizing and sharing the results of functional MRI (Rosset, Spadola & Ratib, 2004; Soares & Alves, 2009), exchange of implantation plans (Treichel et al., 2010), interchange of ECG reports (von Wangenheim et al., 2013) as well as for clinical trial applications in neurology (Jacobs et al., 2010) and oncology (Clunie, 2007).

References

Bender D., Duane B., Kamran S. 2013. HL7 FHIR: An Agile and RESTful approach to healthcare information exchange. In: *Proceedings of the 26th IEEE International Symposium on Computer-Based Medical Systems*. DOI: 10.1109/cbms.2013.6627810.

Brent J. Liu, Anh Le, H.K. Huang. 2015. Integration of CAD and PACS. In: Qiang Li RMN ed. *Computer-Aided Detection and Diagnosis in Medical Imaging*. Imaging in medical diagnosis and therapy. CRC Press, 317–326.

BusinessWire. 2013. Nuance Announces Faster, More Accurate PowerScribe 360 Platform. *Available at http://www.businesswire.com/news/home/20131202005033/en/Nuance-Announces-Faster-Accurate-PowerScribe-360-Platform* (accessed November 17, 2015).

Clunie D. 2000. *DICOM Structured Reporting*. PixelMed Publishing.

Clunie DA. 2007. DICOM Structured Reporting and Cancer Clinical Trials Results. *Cancer informatics* 4:33–56.

Clunie D. 2015a.DICOM Validator - dciodvfy. *Available at http://www.dclunie.com/dicom3tools/dciodvfy.html* (accessed November 22, 2015).

Clunie D. 2015b. DicomSRValidator. *Available at http://www.dclunie.com/pixelmed/software/javadoc/com/pixelmed/validate/DicomSRValidator.html* (accessed November 22, 2015).

Dean Bidgood W Jr. 1998. Clinical importance of the DICOM structured reporting standard. *The international journal of cardiovascular imaging* 14:307–315. DOI: 10.1023/A:1006073709957.

Dolin RH., Alschuler L., Boyer S., Beebe C., Behlen FM., Biron PV., Shabo Shvo A. 2006. HL7 Clinical Document Architecture, Release 2. *Journal of the American Medical Informatics Association: JAMIA* 13:30–39. DOI: 10.1197/jamia.M1888.

Goossen W., Langford LH. 2014. Exchanging care records using HL7 V3 care provision messages. *Journal of the American Medical Informatics Association: JAMIA* 21:e363–8. DOI: 10.1136/amiajnl-2013-002264.

Health Level Seven® International. 2015. Fast Healthcare Interoperability Resources (FHIR). *Available at http://www.hl7.org/FHIR/* (accessed November 17, 2015).
Imaging Technology News. 2013. PACSgear Introduces ModLink to Transfer Structured Reports Into PowerScribe 360. *Available at http://www.itnonline.com/content/pacsgear-introduces-modlink-transfer-structured-reports-powerscribe-360* (accessed November 17, 2015).
Jacobs C., Ma K., Moin P., Liu B. 2010. An automatic quantification system for MS lesions with integrated DICOM structured reporting (DICOM-SR) for implementation within a clinical environment. In: Liu BJ, Boonn WW eds. *Medical Imaging 2010: Advanced PACS-based Imaging Informatics and Therapeutic Applications*. SPIE Proceedings. SPIE, 76280K–76280K–8. DOI: 10.1117/12.844072.
Loef C., Truyen R. 2005. Evidence and Diagnostic Reporting in the IHE Context1. *Academic radiology* 12:620–625. DOI: 10.1016/j.acra.2005.01.020.
MModal. 2014. Fluency for Image Reporting: Intelligence-Driven Documentation and Workflow Management.
Mwogi TS., Biondich PG., Grannis SJ. 2014. An Evaluation of Two Methods for Generating Synthetic HL7 Segments Reflecting Real-World Health Information Exchange Transactions. *AMIA ... Annual Symposium proceedings / AMIA Symposium. AMIA Symposium* 2014:1855–1863.
National Electrical Manufacturers Association (NEMA). 2016. C SR to CDA Imaging Report Transformation Guide. In: *DICOM PS3.20 - Imaging Reports using HL7 Clinical Document Architecture*.
Noumeir R. 2006. Benefits of the DICOM structured report. *Journal of digital imaging : the official journal of the Society for Computer Applications in Radiology* 19:295–306. DOI: 10.1007/s10278-006-0631-7.
Rasmussen LV., Kiefer RC., Mo H., Speltz P., Thompson WK., Jiang G., Pacheco JA., Xu J., Zhu Q., Denny JC., Montague E., Pathak J. 2015. A Modular Architecture for Electronic Health Record-Driven Phenotyping. *AMIA Joint Summits on Translational Science proceedings AMIA Summit on Translational Science* 2015:147–151.
Rosset A., Spadola L., Ratib O. 2004. OsiriX: an open-source software for navigating in multidimensional DICOM images. *Journal of digital imaging : the official journal of the Society for Computer Applications in Radiology* 17:205–216. DOI: 10.1007/s10278-004-1014-6.
Soares J., Alves V. 2009. Functional Magnetic Resonance Imaging Data Manipulation - A new approach. In: Dössel O, Schlegel WC eds. *World Congress on Medical Physics and Biomedical Engineering, September 7 - 12, 2009, Munich, Germany*. IFMBE Proceedings. Berlin, Heidelberg: Springer Berlin Heidelberg, 36–39. DOI: 10.1007/978-3-642-03904-1_10.
Talati RK., Dunkin J., Parikh S., Moore WH. 2013. Current methods of monitoring radiation exposure from CT. *Journal of the American College of Radiology: JACR* 10:702–707. DOI: 10.1016/j.jacr.2013.03.002.
The Cancer Imaging Archive (TCIA). 2015.TCGA-LUSC - The Cancer Imaging Archive (TCIA) Public Access - Cancer Imaging Archive Wiki. *Available at https://wiki.cancerimagingarchive.net/display/Public/TCGA-LUSC* (accessed November 17, 2015).
Treichel T., Liebmann P., Burgert O., Gessat M. 2010. Applicability of DICOM structured reporting for the standardized exchange of implantation plans. *International journal of computer assisted radiology and surgery* 5:1–9. DOI: 10.1007/s11548-009-0375-1.
von Wangenheim A., Barcellos CL Jr., Andrade R., de Carlos Back Giuliano I., Borgatto AF., de Andrade DF. 2013. Implementing DICOM structured reporting in a large-scale telemedicine network. *Telemedicine journal and e-health: the official journal of the American Telemedicine Association* 19:535–541. DOI: 10.1089/tmj.2012.0103.
