# Supplementary material for: DICOM for quantitative imaging biomarker development: a standards based approach to sharing clinical data and structured PET/CT analysis results in head and neck cancer research
Source: PeerJ. 2016 May 24;4:e2057. doi: 10.7717/peerj.2057 (PMC4888317; doi:10.7717/peerj.2057)
Supplement: Appendix S2 [file peerj-04-2057-s002.pdf]

Appendix 2: QIICR Iowa Head and Neck Clinical Data DICOM SR Template

Table of Contents

Document History
TID QIICR_2000. Clinical Data Report
TID QIICR_2002. Biopsy
TID QIICR_2003. Surgical Procedure for Head and Neck Cancer
TID QIICR_2004. Radiotherapy Procedure
TID QIICR_2005. Chemotherapy Procedure
TID QIICR_2006. Tumor Pathology Results
TID QIICR_2007. Cervical Lymph Node Group
TID QIICR_2008. Diabetes Problem Properties
CID QIICR_2001. Racial Group
CID QIICR_2002. Performed
CID QIICR_2003. Alcohol Intake
CID QIICR_2004. Tobacco Chewing
CID QIICR_2005. Clinical Stage
CID QIICR_2006. TNM T Stage
CID QIICR_2007. TNM N Stage
CID QIICR_2008. TNM M Stage
CID QIICR_2009. Malignancy History
CID QIICR_2010. Extra-capsular Extension of Nodal Tumor
CID QIICR_2011. Followup Status
CID QIICR_2012. Cause of Death
CID QIICR_2013. Location of Recurrent Tumor
CID QIICR_2014. Extent of Resection
CID QIICR_2015. Antineoplastic Agent
CID QIICR_2016. Histologic Grade
CID QIICR_2017. Malignancy Type
CID QIICR_2018. Tumor Margin Status
CID QIICR_2019. Perineural Invasion
CID QIICR_2020. Vascular Invasion
CID QIICR_2021. Lymph Node Group
CID QIICR_2022. Same or Different Side
CID 230. Yes-No
CID 3722. Diabetic Therapy
CID 3724. Smoking History
CID 3769. Concern Types
CID 7455. Sex
CID 7601. Head and Neck Cancer Anatomic Sites
X. 99PMP Controlled Terminology Definitions

List of Tables

TID QIICR_2000. Measurement Report
TID QIICR_2002. Biopsy
TID QIICR_2003. Surgical Procedure for Head and Neck Cancer
TID QIICR_2004. Radiotherapy Procedure
TID QIICR_2005. Chemotherapy Procedure
TID QIICR_2006. Tumor Pathology Results
TID QIICR_2007. Cervical Lymph Node Group
TID QIICR_2008. Diabetes Problem Properties
CID QIICR_2001. Racial Group
CID QIICR_2002. Performed
CID QIICR_2003. Alcohol Intake
CID QIICR_2004. Tobacco Chewing
CID QIICR_2005. Clinical Stage
CID QIICR_2006. TNM T Stage
CID QIICR_2007. TNM N Stage
CID QIICR_2008. TNM M Stage
CID QIICR_2009. Malignancy History
CID QIICR_2010. Extra-capsular Extension of Nodal Tumor
CID QIICR_2011. Followup Status
CID QIICR_2012. Cause of Death
CID QIICR_2013. Location of Recurrent Tumor
CID QIICR_2014. Extent of Resection
CID QIICR_2015. Antineoplastic Agent
CID QIICR_2016. Histologic Grade
CID QIICR_2017. Malignancy Type
CID QIICR_2018. Tumor Margin Status
CID QIICR_2019. Perineural Invasion
CID QIICR_2020. Vascular Invasion
CID QIICR_2021. Lymph Node Group
CID QIICR_2022. Same or Different Side
CID 230. Yes-No
CID 3722. Diabetic Therapy
CID 3724. Smoking History
CID 3769. Concern Types
CID 7455. Sex
CID 7601. Head and Neck Cancer Anatomic Sites
X-n. 99PMP Controlled Terminology Definitions

Document History

Document Version	Date	Content
01	2015/03/01	First draft delivered to Iowa for clinical data release to TCIA
02	2015/10/07	Correct typos, add SCT and UMLS code links for use when comparing with ASCO HL7 CDA COTPS, add list of 99PMP codes used with placeholders for definitions and hyperlink from uses.

TID QIICR_2000 Clinical Data Report

This root template that encodes the clinical data for a patient.

Type: **Extensible**

Order: **Non-Significant**

Root: **Yes**

Table TID QIICR_2000. Measurement Report

	NL	Rel with Parent	VT	Concept Name	VM	Req Type	Condition	Value Set Constraint
1			CONTAINER	EV (R-42BAB, SRT, "Summary Clinical Document")	1	M		Root node
2	>	HAS CONCEPT MOD	INCLUDE	DTID 1204 "Language of Content Item and Descendants"	1	M		
3	>	CONTAINS	CONTAINER	EV (121118, DCM, "Patient Characteristics")	1	M		
4	>>	CONTAINS	DATE	EV (121031, DCM, "Subject Birth Date")	1	U		
5	>>	CONTAINS	CODE	EV (121032, DCM, "Subject Sex")	1	U		DCID 7455 “Sex”
6	>>	CONTAINS	NUM	EV (8302-2, LN, "Patient Height")	1	U		UNITS = EV (cm, UCUM, "cm")
7	>>	CONTAINS	NUM	EV (29463-7, LN, "Patient Weight")	1	U		UNITS = EV (kg, UCUM, "kg")
8	>>	CONTAINS	CODE	EV (S-0004D, SRT, "Racial group")	1	U		DCID QIICR_2001 “Racial Group”
9	>>	CONTAINS	CODE	EV (S-00045, SRT, "Hispanic")	1	U		DCID 230 “Yes-No”
10	>	CONTAINS	CONTAINER	EV (11450-4, LN, "Problem List")	1	M		
11	>>	CONTAINS	INCLUDE	DTID QIICR_2008 “Diabetes Problem Properties”	1	U		
12	>	CONTAINS	CONTAINER	EV (29762-2, LN, "Social History")	1	M		
13	>>	CONTAINS	CODE	EV (F-93109, SRT, "Tobacco Smoking Behavior")	1	U		DCID 3724 “Smoking History”
14	>>	CONTAINS	CODE	EV (F-02573, SRT, "Alcohol consumption")	1	U		DCID QIICR_2003 “Alcohol Intake”

15	>>	CONTAINS	CODE	EV (F-0434C, SRT, "Details of tobacco chewing")	1	U		DCID QIICR_2004 “Tobacco Chewing”
16	>	CONTAINS	CONTAINER	EV (G-E395, SRT, "Tumor Staging")	1	M		
17	>>	CONTAINS	CODE	EV (R-100D9, SRT, "Primary tumor site")	1	U		DCID 7601 “Head and Neck Cancer Anatomic Sites”
18	>>	CONTAINS	CODE	EV (R-00443, SRT, "Tumor stage finding")	1	U		DCID QIICR_2005 “Clinical Stage”
19	>>	CONTAINS	CONTAINER	EV (F-005C4, SRT, "TNM Category")	1	U		
20	>>>	CONTAINS	CODE	EV (G-F150, SRT, "T Stage")	1	U		DCID QIICR_2006 “TNM T Stage”
21	>>>	CONTAINS	CODE	EV (R-40030, SRT, "N Stage")	1	U		DCID QIICR_2007 “TNM N Stage”
22	>>>	CONTAINS	CODE	EV (R-40031, SRT, "M Stage")	1	U		DCID QIICR_2008 “TNM M Stage”
23	>	CONTAINS	CONTAINER	EV (G-03E7, SRT, "Past medical history")	1	M		
24	>>	CONTAINS	CODE	EV (P0-099EB, SRT, "History of radiation therapy")	1	U		DCID QIICR_2002 “Performed”
25	>>	CONTAINS	CODE	EV (G-0133, SRT, "History of malignant neoplasm")	1	U		DCID QIICR_2009 “Malignancy History”
26	>	CONTAINS	CONTAINER	EV (P0-00002, SRT, "Diagnostic Procedure")	1	M		
27	>>	CONTAINS	INCLUDE	DTID QIICR_2002 “Biopsy”	1	U		
28	>	CONTAINS	CONTAINER	EV (P0-0000E, SRT, "Therapeutic Procedure")	1	M		
29	>>	CONTAINS	INCLUDE	DTID QIICR_2003 “Surgical Procedure for Head and Neck Cancer”	1	U		
30	>>	CONTAINS	INCLUDE	DTID QIICR_2004 “Radiotherapy Procedure”	1	U		
31	>>	CONTAINS	INCLUDE	DTID QIICR_2005 “Chemotherapy Procedure”	1	U		
32	>	CONTAINS	CONTAINER	EV (300015, 99PMP, "Pathology of original tumor")	1	M		
33	>>	CONTAINS	INCLUDE	DTID QIICR_2006 “Tumor Pathology Results”	1	U		

34	>>	CONTAINS	CONTAINER	EV (P1-65320, SRT, "Excision of cervical lymph nodes group")	1	M		
35	>>>	CONTAINS	INCLUDE	DTID QIICR_2007 “Cervical Lymph Node Group”	1	U		
36	>>>	CONTAINS	CODE	EV (F-004ED, SRT, "Status of extra-capsular extension of nodal tumor")	1	U		DCID QIICR_2010 “Extra-capsular Extension of Nodal Tumor”
37	>>>	CONTAINS	TEXT	EV (121106, DCM, "Comment")	1	U		
38	>	CONTAINS	CONTAINER	EV (C0679250, UMLS, "Disease Outcome")	1	M		
39	>>	CONTAINS	DATE	EV (C3694716, UMLS, "Follow-up visit date")	1	U		
40	>>	CONTAINS	CODE	EV (F-00F54, SRT, "Followup status")	1	U		DCID QIICR_2011 “Followup Status”
41	>>	CONTAINS	DATE	EV (F-04922, SRT, "Date of death")	1	U		
42	>>	CONTAINS	CODE	EV (F-03E6D, SRT, "Cause of death")	1	U		DCID QIICR_2012 “Cause of Death”
43	>>	CONTAINS	CODE	EV (300011, 99PMP, "Post-radiotherapy treatment")	1	U		DCID 230 “Yes-No”
44	>>	CONTAINS	DATE	EV (63944-3, LN, "Date of cancer recurrence")	1	U		
45	>>	CONTAINS	DATE	EV (300012, 99PMP, "Date of 2nd primary")	1	U		
46	>>	CONTAINS	CODE	EV (300013, 99PMP, "Location of first recurrence")	1	U		DCID QIICR_2013 “Location of Recurrent Tumor”
47	>>	CONTAINS	CONTAINER	EV (300016, 99PMP, "Pathology of recurrent tumor")	1	U		
48	>>>	CONTAINS	INCLUDE	DTID QIICR_2006 “Tumor Pathology Results”	1	M		

Content Item Descriptions

Row 2	Always (121049, DCM, "Language of Content Item and Descendants") = (eng, RFC3066, "English")<br>>HAS CONCEPT MOD (121046, DCM, "Country of Language") = (US, ISO3166_1, "United States")
Row 5	Only (M, DCM, "Male") and (F, DCM, "Female") are used.

Rows 9, 43	(R-0038A, SRT, "Undetermined") from CID 230 “Yes-No” is not used.
Rows 12, 13	Based on TID 3802 Cardiovascular Patient History, rows 12 and 15.
Row 14	TID 3802 Row 14 has this as one of the concepts for a TEXT value drawn from CID_3774; we use a CODE instead
Row 15	Nothing in DICOM yet, except as (C-F3310, SRT, "Chewing tobacco") in CID 6089 Substances invoked in DTID 9002 “Medication, Substance, Environmental Exposure” in TID 9007 General Relevant Patient Information
Row 25	When no specific information is available, will be same concept for name and value (G-0133 = G-0133).

TID QIICR_2002 Biopsy

This template encodes information about a biopsy procedure.

Type: **Extensible**

Order: **Non-Significant**

Root: **No**

Table TID QIICR_2002. Biopsy

	NL	Rel with Parent	VT	Concept Name	VM	Req Type	Condition	Value Set Constraint
1			CONTAINER	EV (P1-03100, SRT, "Biopsy")	1-n	M		
2	>	CONTAINS	DATE	EV (F-05045, SRT, "Date of procedure")	1	U		
3	>	CONTAINS	TEXT	EV (F-04956, SRT, "Biopsy Site")	1	U		

TID QIICR_2003 Surgical Procedure for Head and Neck Cancer

This template encodes information about a surgical procedure for head and neck cancer.

Type: **Extensible**

Order: **Non-Significant**

Root: **No**

Table TID QIICR_2003. Surgical Procedure for Head and Neck Cancer

	NL	Rel with Parent	VT	Concept Name	VM	Req Type	Condition	Value Set Constraint
1			CONTAINER	EV (P0-009C3, SRT, "Surgical Procedure")	1-n	M		
2	>	CONTAINS	DATE	EV (F-05045, SRT, "Date of procedure")	1	U		
3	>	CONTAINS	TEXT	EV (C0807506, UMLS, "Procedure Description")	1	U		
4	>	CONTAINS	CODE	EV (300001, 99PMP, "Resection of primary tumor")	1	U		DCID QIICR_2014 “Extent of Resection”
5	>	CONTAINS	CODE	EV (P1-65325, SRT, "Block dissection of cervical lymph nodes")	1	U		DCID QIICR_2002 “Performed”

Content Item Descriptions

Row 4	EV (P1-03002, SRT, "Complete excision") is not quite right, because strictly speaking it applies to the organ

TID QIICR_2004 Radiotherapy Procedure

This template encodes information about a radiotherapy procedure.

Type: **Extensible**

Order: **Non-Significant**

Root: **No**

Table TID QIICR_2004. Radiotherapy Procedure

	NL	Rel with Parent	VT	Concept Name	VM	Req Type	Condition	Value Set Constraint
1			CONTAINER	EV (P5-C0000, SRT, "Radiotherapy Procedure")	1-n	M		
2	>	CONTAINS	DATE	EV (F-04C2B, SRT, "Date treatment started")	1	U		
3	>	CONTAINS	DATE	EV (F-04C2C, SRT, "Date treatment stopped")	1	U		
4	>	CONTAINS	NUM	EV (R-007B0, SRT, "Total radiation dose delivered")	1	U		UNITS = EV (Gy, UCUM, "Gy")
5	>	CONTAINS	NUM	EV (300002, 99PMP, "Radiation dose per fraction")	1	U		UNITS = EV (Gy, UCUM, "Gy")
6	>	CONTAINS	TEXT	EV (C0807506, UMLS, "Procedure Description")	1	U		

TID QIICR_2005 Chemotherapy Procedure

This template encodes information about a chemotherapy procedure.

Type: **Extensible**

Order: **Non-Significant**

Root: **No**

Table TID QIICR_2005. Chemotherapy Procedure

	NL	Rel with Parent	VT	Concept Name	VM	Req Type	Condition	Value Set Constraint
1			CONTAINER	EV (P0-0058E, SRT, "Chemotherapy")	1-n	M		
2	>	CONTAINS	DATE	EV (F-04C2B, SRT, "Date treatment started")	1	U		
3	>	CONTAINS	DATE	EV (F-04C2C, SRT, "Date treatment stopped")	1	U		
4	>	CONTAINS	CODE	EV (F-618AA, SRT, "Antineoplastic agent")	1-3	U		DCID QIICR_2015 “Antineoplastic Agent”

TID QIICR_2006 Tumor Pathology Results

This template encodes information about the pathology of a tumor.

Type: **Extensible**

Order: **Non-Significant**

Root: **No**

Table TID QIICR_2006. Tumor Pathology Results

	NL	Rel with Parent	VT	Concept Name	VM	Req Type	Condition	Value Set Constraint
1			CONTAINER	EV (111468, DCM, "Pathology Results")	1	M		
2	>	CONTAINS	CODE	EV (111042, DCM, "Pathology")	1	U		EV (M-80703, SRT, "Squamous Cell Carcinoma")
3	>>	HAS PROPERTIES	CODE	EV (F-02900, SRT, "Histological grade finding")	1	U		DCID QIICR_2016 “Histologic Grade”
4	>>	HAS PROPERTIES	CODE	EV (111388, DCM, "Malignancy Type")	1	U		DCID QIICR_2017 “Malignancy Type”
5	>>	HAS PROPERTIES	CODE	EV (R-00274, SRT, "Tumor margin status")	1	U		DCID QIICR_2018 “Tumor Margin Status”
6	>>	HAS PROPERTIES	CODE	EV (F-0369E, SRT, "Perineural invasion finding")	1	U		DCID QIICR_2019 “Perineural Invasion”
7	>>	HAS PROPERTIES	CODE	EV (R-0026E, SRT, "Status of vascular invasion by tumor")	1	U		DCID QIICR_2020 “Vascular Invasion”

Content Item Descriptions

Row 1	This template uses TID 4207 Breast Imaging Pathology Results as model.
Row 3	The values are the same as DCID 6070, which is described as Bloom-Richardson Histologic Grade, which is breast-specific, though the code values, which are re-used here, are just ordinary AJCC.
Row 5	TID 4207 (currently) uses (111471, DCM, "Involved") instead of (G-8DA4, SRT, "Surgical margin involved by tumor"), and (111470, DCM, "Uninvolved") instead of (M-09400, SRT, "Surgical margin uninvolved by tumor").
Row 7	SNOMED "vascular" concept has children of large and small vessels, and latter includes lymphatic vessels.

TID QIICR_2007 Cervical Lymph Node Group

This template encodes information about a cervical lymph node group.

Type: **Extensible**

Order: **Non-Significant**

Root: **No**

Table TID QIICR_2007. Cervical Lymph Node Group

	NL	Rel with Parent	VT	Concept Name	VM	Req Type	Condition	Value Set Constraint
1			CODE	EV (T-C4207, SRT, "Cervical lymph node group")	1-n	M		DCID QIICR_2021 “Lymph Node Group”
2	>	HAS CONCEPT MOD	CODE	EV (R-400D5, SRT, "Sidedness")	1	U		DCID QIICR_2022 “Same or Different Side”
3	>	HAS PROPERTIES	NUM	EV (111473, DCM, "Number of nodes removed")	1	U		UNITS = EV ({nodes}, UCUM, "nodes")
4	>	HAS PROPERTIES	NUM	EV (111474, DCM, "Number of nodes positive")	1	U		UNITS = EV ({nodes}, UCUM, "nodes")

TID QIICR_2008 Diabetes Problem Properties

This template encodes information diabetes mellitus information for a problem list.

Type: **Extensible**
Order: **Significant**

Table TID QIICR_2008. Diabetes Problem Properties

	NL	Rel with Parent	VT	Concept Name	VM	Req Type	Condition	Value Set Constraint
1			CONTAINER	EV (121430, DCM, "Concern")	1	M		
2	>	CONTAINS	CODE	EV (F-01000, SRT, "Problem")	1	M		EV (G-023F, SRT, "History of Diabetes mellitus")
3	>	CONTAINS	CODE	EV (P0-0000E, SRT, "Therapy")	1-n	U		DCID 3722 “Diabetic Therapy”

Content Item Descriptions

Rows 1,2,3	Correspond to rows 1, 4 (Problem) and 12 (Therapy) of TID 3829 "Problem Properties", and only (F-01000, SRT, "Problem) from CID 3769 “Concern Types” is used in row 4

CID QIICR_2001 Racial Group

Type: **Extensible**
Version: **20150228**

Table CID QIICR_2001. Racial Group

Coding Scheme Designator	Code Value	Code Meaning	SNOMED-CT Concept ID	UMLS Concept Unique ID
SRT	S-0004E	African race	413464008	C0027567
SRT	S-00051	Asian race	413582008	C0078988
SRT	S-0003D	Caucasian race	413773004	C0007457

SRT	S-0004F	American Indian race	413491005	C0002460

CID QIICR_2002 Performed

Type: **Extensible**
Version: **20150228**

Table CID QIICR_2002. Performed

Coding Scheme Designator	Code Value	Code Meaning	SNOMED-CT Concept ID	UMLS Concept Unique ID
SRT	R-42514	Performed	398166005	C0884358
SRT	R-4135B	Not performed	262008008	C0445106

CID QIICR_2003 Alcohol Intake

Type: **Extensible**
Version: **20150228**

Table CID QIICR_2003. Alcohol Intake

Coding Scheme Designator	Code Value	Code Meaning	SNOMED-CT Concept ID	UMLS Concept Unique ID
SRT	F-60018	Alcohol intake above recommended sensible limits	160592001	C0560219
SRT	F-60019	Alcohol intake within recommended sensible limits	160593006	C0560220
SRT	R-40775	None	260413007	C0549184

CID QIICR_2004 Tobacco Chewing

Type: **Extensible**
Version: **20150228**

Table CID QIICR_2004. Tobacco Chewing

Coding Scheme Designator	Code Value	Code Meaning	SNOMED-CT Concept ID	UMLS Concept Unique ID
SRT	S-32060	Chews tobacco	81703003	C0241410
SRT	F-9321B	Ex-tobacco chewer	228513009	C0454023
SRT	F-93219	Does not chew tobacco	228511006	C0454021

CID QIICR_2005 Clinical Stage

Type: **Extensible**
Version: **20150228**

Table CID QIICR_2005. Clinical Stage

Coding Scheme Designator	Code Value	Code Meaning	SNOMED-CT Concept ID	UMLS Concept Unique ID

SRT	G-E100	Clinical Stage I	13104003	C0205564
SRT	G-E200	Clinical Stage II	60333009	C0205571
SRT	G-E300	Clinical Stage III	50283003	C0205578
SRT	G-E400	Clinical Stage IV	2640006	C0205585
SRT	G-E410	Clinical Stage IV A	71725004	C0205586
SRT	G-E420	Clinical Stage IV B	1523005	C0205587
SRT	G-E430	Clinical Stage IV C	33177002	C0205588

CID QIICR_2006 TNM T Stage

Type: **Extensible**
Version: **20150228**

Table CID QIICR_2006. TNM T Stage

Coding Scheme Designator	Code Value	Code Meaning	SNOMED-CT Concept ID	UMLS Concept Unique ID
SRT	G-F152	Tumor Stage T0	58790005	C0475371
SRT	G-F153	Tumor Stage T1	23351008	C0475372
SRT	G-F158	Tumor Stage T1a	261646003	C0475383
SRT	G-F15B	Tumor Stage T1b	261649005	C0475385
SRT	G-F154	Tumor Stage T2	67673008	C0475373
SRT	G-F15D	Tumor Stage T2a	261651009	C0475387
SRT	G-F15E	Tumor Stage T2b	261652002	C0475388
SRT	G-F155	Tumor Stage T3	14410001	C0475374
SRT	G-F16D	Tumor Stage T3a	261654001	C0475390
SRT	G-F16E	Tumor Stage T3b	261655000	C0475391
SRT	G-F156	Tumor Stage T4	65565005	C0475751
SRT	G-F176	Tumor Stage T4a	261659006	C0475395
SRT	G-F177	Tumor Stage T4b	261660001	C0475396
SRT	G-F157	Tumor Stage TX	67101007	C0332377

CID QIICR_2007 TNM N Stage

Type: **Extensible**
Version: **20150228**

Table CID QIICR_2007. TNM N Stage

Coding Scheme Designator	Code Value	Code Meaning	SNOMED-CT Concept ID	UMLS Concept Unique ID
SRT	G-F160	Node Stage N0	62455006	C0441959
SRT	G-F161	Node Stage N1	53623008	C0441962
SRT	G-F166	Node Stage N1a	277672002	C0456906
SRT	G-F167	Node Stage N1b	277674001	C0456908
SRT	G-F162	Node Stage N2	46059003	C0441960
SRT	G-F17E	Node Stage N2a	261967001	C0445079
SRT	G-F17F	Node Stage N2b	261968006	C0445080
SRT	G-F188	Node Stage N2c	261969003	C0445081
SRT	G-F163	Node Stage N3	5856006	C0441961
SRT	G-F165	Node Stage NX	79420006	C0445085

CID QIICR_2008 TNM M Stage

Type: **Extensible**
Version: **20150228**

Table CID QIICR_2008. TNM M Stage

Coding Scheme Designator	Code Value	Code Meaning	SNOMED-CT Concept ID	UMLS Concept Unique ID
SRT	G-F170	Metastasis Stage M0	30893008	C0445034
SRT	G-F171	Metastasis Stage M1	55440008	C0441971
SRT	G-F175	Metastasis Stage MX	27167007	C0445039

CID QIICR_2009 Malignancy History

Type: **Extensible**
Version: **20150228**

Table CID QIICR_2009. Malignancy History

Coding Scheme Designator	Code Value	Code Meaning	SNOMED-CT Concept ID	UMLS Concept Unique ID
SRT	G-0529	History of malignant neoplasm of head and/or neck	431573004	C2317135
SRT	G-0133	History of malignant neoplasm	266987004	C0455471

SRT	R-FB75F	No history of malignant neoplastic disease	700363003	C3494909

CID QIICR_2010 Extra-capsular Extension of Nodal Tumor

Type: **Extensible**
Version: **20150228**

Table CID QIICR_2010. Extra-capsular Extension of Nodal Tumor

Coding Scheme Designator	Code Value	Code Meaning	SNOMED-CT Concept ID	UMLS Concept Unique ID
SRT	F-004F1	Extra-capsular extension of nodal tumor present	396644006	C1300901
SRT	F-004EF	Extra-capsular extension of nodal tumor absent	396643000	C1300900

CID QIICR_2011 Followup Status

Type: **Extensible**
Version: **20150228**

Table CID QIICR_2011. Followup Status

Coding Scheme Designator	Code Value	Code Meaning	SNOMED-CT Concept ID	UMLS Concept Unique ID
UMLS	C1518340	No evidence of disease		C1518340
SRT	DF-00280	Local disease	74217003	C0277565
UMLS	C3641061	Distant metastases		C3641061
99PMP	300010	Local disease and distant metastases		

CID QIICR_2012 Cause of Death

Type: **Extensible**
Version: **20150228**

Table CID QIICR_2012. Cause of Death

Coding Scheme Designator	Code Value	Code Meaning	SNOMED-CT Concept ID	UMLS Concept Unique ID
SRT	DD-60001	Complication	116223007	C0009566
SRT	DF-00280	Local disease	74217003	C0277565
UMLS	C3641061	Distant metastases		C3641061
99PMP	300010	Local disease and distant metastases		

SRT	DF-00170	Intercurrent disease	88472004	C0277557

CID QIICR_2013 Location of Recurrent Tumor

Type: **Extensible**
Version: **20150228**

Table CID QIICR_2013. Location of Recurrent Tumor

Coding Scheme Designator	Code Value	Code Meaning	SNOMED-CT Concept ID	UMLS Concept Unique ID
SRT	DF-00280	Local disease	74217003	C0277565
SRT	G-A16D	Region	410673009	C0205147
UMLS	C3641061	Distant metastases		C3641061
99PMP	300014	Local and regional		
99PMP	300010	Local disease and distant metastases		

CID QIICR_2014 Extent of Resection

Type: **Extensible**
Version: **20150228**

Table CID QIICR_2014. Extent of Resection

Coding Scheme Designator	Code Value	Code Meaning	SNOMED-CT Concept ID	UMLS Concept Unique ID
SRT	P1-03002	Complete excision	79095000	C0015250
SRT	P1-03001	Partial excision	38829003	C0184908
SRT	R-4135B	Not performed	262008008	C0445106

CID QIICR_2015 Antineoplastic Agent

Type: **Extensible**
Version: **20150228**

Table CID QIICR_2015. Antineoplastic Agent

Coding Scheme Designator	Code Value	Code Meaning	SNOMED-CT Concept ID	UMLS Concept Unique ID
SRT	F-61F04	Cetuximab	409400001	C0995188
SRT	C-15310	Platinum	11996000	C3536919
SRT	C-3013D	Taxane	418965003	C1642388
SRT	C-780F0	5FU	3127006	C0016360

CID QIICR_2016 Histologic Grade

Type: Extensible
Version: 20150228

Table CID QIICR_2016. Histologic Grade

Coding Scheme Designator	Code Value	Code Meaning	SNOMED-CT Concept ID	UMLS Concept Unique ID
SRT	G-F211	Grade 1: well differentiated	54102005	C0475269
SRT	G-F212	Grade 2: moderately differentiated	1663004	C0475270
SRT	G-F213	Grade 3: poorly differentiated	61026006	C0475271
SRT	R-41DC5	Grade 4: undifferentiated	258245003	C0475272

CID QIICR_2017 Malignancy Type

Type: Extensible
Version: 20150228

Table CID QIICR_2017. Malignancy Type

Coding Scheme Designator	Code Value	Code Meaning	SNOMED-CT Concept ID	UMLS Concept Unique ID
SRT	D1-F3502	Carcinoma in situ	109355002	C0007099
UMLS	C1334274	Invasive carcinoma		C1334274

CID QIICR_2018 Tumor Margin Status

Type: Extensible
Version: 20150228

Table CID QIICR_2018. Tumor Margin Status

Coding Scheme Designator	Code Value	Code Meaning	SNOMED-CT Concept ID	UMLS Concept Unique ID
SRT	G-8DA4	Surgical margin involved by tumor	370109009	C0332649
99PMP	300004	Surgical margin close to tumor		
SRT	M-09400	Surgical margin uninvolved by tumor	55182004	C0332648

CID QIICR_2019 Perineural Invasion

Type: Extensible
Version: 20150228

Table CID QIICR_2019. Perineural Invasion

Coding Scheme Designator	Code Value	Code Meaning	SNOMED-CT Concept ID	UMLS Concept Unique ID
SRT	G-F538	Perineural invasion by tumor present	369731000	C1269945
SRT	G-F7A3	Perineural invasion by tumor absent	370051000	C1269954

CID QIICR_2020 Vascular Invasion

Type: **Extensible**
Version: **20150228**

Table CID QIICR_2020. Vascular Invasion

Coding Scheme Designator	Code Value	Code Meaning	SNOMED-CT Concept ID	UMLS Concept Unique ID
SRT	R-002A7	Vascular invasion by tumor present	372287009	C1318569
SRT	G-F519	Vascular invasion by tumor absent	127494000	C1264755

CID QIICR_2021 Lymph Node Group

Type: **Extensible**
Version: **20150228**

Table CID QIICR_2021. Lymph Node Group

Coding Scheme Designator	Code Value	Code Meaning	SNOMED-CT Concept ID	UMLS Concept Unique ID
99PMP	300005	Level I - Submental and submandibular lymph node group		
SRT	T-C420B	Level II - Upper jugular lymph node group	245261007	C0447165
SRT	T-C420C	Level III - Middle jugular lymph node group	245262000	C0447167
SRT	T-C420D	Level IV - Lower jugular lymph node group	245263005	C0447166
SRT	T-C420E	Level V - Posterior triangle cervical lymph node group	245264004	C0447168
99PMP	300006	Cervical lymph node outside level I through V		

CID QIICR_2022 Same or Different Side

Type: **Extensible**
Version: **20150228**

Table CID QIICR_2022. Same or Different Side

Coding Scheme Designator	Code Value	Code Meaning	SNOMED-CT Concept ID	UMLS Concept Unique ID
SRT	R-40356	Ipsilateral	255208005	C0441989
SRT	R-40357	Contralateral	255209002	C0441988

CID 230 Yes-No

Type: **Non-Extensible**
Version: **20060613**

Table CID 230. Yes-No

Coding Scheme Designator	Code Value	Code Meaning	SNOMED-CT Concept ID	UMLS Concept Unique ID
SRT	R-0038D	Yes	373066001	C1298907
SRT	R-00339	No	373067005	C1298908
SRT	R-0038A	Undetermined	373068000	C3536725

CID 3722 Diabetic Therapy

Type: **Extensible**
Version: **20141103**

Table CID 3722. Diabetic Therapy

Coding Scheme Designator	Code Value	Code Meaning	SNOMED-CT Concept ID	UMLS Concept Unique ID
SRT	F-02F14	Diabetic on Dietary Treatment	170745003	C0421246
SRT	F-02F15	Diabetic on Oral Treatment	170746002	C0421247
SRT	F-02F16	Diabetic on Insulin	170747006	C0421248

Note

In prior editions, this Context Group included the NCDR 2.0b codes as the primary set. These have been replaced with equivalent SNOMED codes.

CID 3724 Smoking History

Type: **Extensible**
Version: **20070827**

Table CID 3724. Smoking History

Coding Scheme Designator	Code Value	Code Meaning	NCDR [2.0b] Equivalent	SNOMED-CT Concept ID
SRT	F-9321F	No History of Smoking	38-0	266919005

SRT	S-32000	Current Smoker	38-1	77176002
SRT	S-32070	Former Smoker	38-2	8517006

Note

In prior editions, this Context Group included the NCDR 2.0b codes as the primary set. These have been replaced with equivalent SNOMED codes.

CID 3769 Concern Types

Type: **Extensible**
Version: **20070827**

Table CID 3769. Concern Types

Coding Scheme Designator	Code Value	Code Meaning	SNOMED-CT Concept ID	UMLS Concept Unique ID
SRT	F-04BA9	Complaint	409586006	C0277786
SRT	DF-00000	Disease	64572001	C0012634
SRT	R-005AE	Finding	404684003	C0037088
SRT	R-005E0	Finding reported by patient/informant	418799008	C1689949
SRT	F-03E55	Functional performance and activity	248536006	C0424866
SRT	F-01000	Problem	55607006	C0033213

CID 7455 Sex

This Context Group includes terms for the finding of sex of a subject for clinical purposes, such as selection of sex-based growth metrics.

Type: **Non-Extensible**
Version: **20040112**

Table CID 7455. Sex

Coding Scheme Designator	Code Value	Code Meaning	Patient's Sex (0010,0040) Equivalent
DCM	M	Male	M
DCM	F	Female	F
DCM	U	Unknown sex	
DCM	MP	Male Pseudohermaphrodite	
DCM	FP	Female Pseudohermaphrodite	
DCM	H	Hermaphrodite	

DCM	MC	Male changed to Female	
DCM	FC	Female changed to Male	
DCM	121104	Ambiguous sex	
DCM	121102	Other sex	
DCM	121103	Undetermined sex	O

Note

1. These terms are distinct from the gender of a subject for administrative purposes, although the default value for clinical sex is often based on the administrative gender (e.g., see TID 1007 "Subject Context, Patient"). The administrative value "O" from Patient's Sex (0010,0040) maps by default to "undetermined" for clinical purposes.
2. This Context Group in a prior edition of the Standard included codes improperly attributed to ISO 5218.
3. These terms are derived from the terminology and codes for sex in ASTM E1633-02a "Standard Specification for Coded Values Used in the Electronic Health Record."

CID 7601 Head and Neck Cancer Anatomic Sites

Type: **Extensible**
Version: **20150106**

Table CID 7601. Head and Neck Cancer Anatomic Sites

Coding Scheme Designator	Code Value	Code Meaning	SNOMED-CT Concept ID	UMLS Concept Unique ID
SRT	T-53131	base of tongue	7283002	C0226958
SRT	T-51305	buccal mucosa	16811007	C1578559
SRT	T-51200	floor of mouth	36360002	C0026638
SRT	T-24440	glottis	1307006	C0017681
SRT	T-55300	hypopharynx	81502006	C0020629
SRT	T-24100	larynx	4596009	C0023078
SRT	T-C5140	lingual tonsil	2048000	C0229871
SRT	T-52000	lip	48477009	C0023759
SRT	T-D07CB	lower alveolar ridge	288546009	C0222755
SRT	T-22100	maxillary sinus	15924003	C0024957
SRT	T-21301	nasal cavity	279549004	C0027423
SRT	T-23000	nasopharynx	71836000	C0027442
SRT	T-51004	oral cavity	74262004	C0226896

SRT	T-C5000	oropharyngeal tonsil (waldeyer's ring)	17861009	C0459892
SRT	T-55200	oropharynx	31389004	C0521367
SRT	T-C5100	palatine tonsil	75573002	C0040421
SRT	T-51130	palatine uvula	26140008	C0042173
SRT	T-22000	paranasal sinus	2095001	C0030471
SRT	T-C5300	pharyngeal tonsil (adenoid)	55940004	C0001428
SRT	T-55320	pyriform sinus	6217003	C0227170
SRT	T-51600	retromolar trigone	85816001	C0226920
SRT	T-61007	salivary gland	385294005	C0036098
SRT	T-24454	supraglottis	119255006	C0225574
SRT	T-53000	tongue	21974007	C0040408
SRT	T-C5001	tonsil and adenoid	303337002	C0580788
SRT	T-C5330	tubal tonsil	21058000	C0229883
UMLS	C0221297	unknown primary neoplasia site		C0221297
SRT	T-51130	uvula	26140008	C0042173

X 99PMP Controlled Terminology Definitions

Table X-n. 99PMP Controlled Terminology Definitions

Code Value	Code Meaning	Definition	Notes
300001	Resection of primary tumor		
300002	Radiation dose per fraction		
300004	Surgical margin close to tumor		
300005	Level I - Submental and submandibular lymph node group		
300006	Cervical lymph node outside level I through V		
300010	Local disease and distant metastases		
300011	Post-radiotherapy treatment		
300012	Date of 2nd primary		
300013	Location of first recurrence		

300014	Local and regional		
300015	Pathology of original tumor		
300016	Pathology of recurrent tumor		
