# Supplementary material for: DICOM for quantitative imaging biomarker development: a standards based approach to sharing clinical data and structured PET/CT analysis results in head and neck cancer research
Source: PeerJ. 2016 May 24;4:e2057. doi: 10.7717/peerj.2057 (PMC4888317; doi:10.7717/peerj.2057)
Supplement: Appendix S3 [file peerj-04-2057-s003.pdf]

## Appendix 3: QIICR PET SUV measurements of interest and their corresponding codes

Research codes corresponding to the measurements match those in the source code of the QuantitativeIndicesCLI tool performing the calculations[1]. Recently an error has been discovered in the UCUM codes specified in the current DICOM standard for SUVbw units, specifically "g/ml{SUVbw}" should be used in place of "{SUVbw}g/ml", due to the UCUM lexical construction rules. A correction to the DICOM standard has since been approved (CP 1535 [ http://www.dclunie.com/dicom-status/status.html#CP1535 ]). The DICOM SR and RWVM files in the repository need to be updated with corrected codes for the units. The numeric values are not affected.

| Measurement type research code | Measurement code | Modifier relationship | Modifier code | Units code |
|---|---|---|---|---|
| Mean_s | (126401,DCM,SUVbw) | (121401,DCM,Derivation) | (R-00317,SRT,"Mean") | ({SUVbw}g/ml,UCUM,"Standardized Uptake Value body weight") |
| Min_s | (126401,DCM,SUVbw) | (121401,DCM,Derivation) | (R-404FB,SRT,"Minimum") | — " — |
| Max_s | (126401,DCM,SUVbw) | (121401,DCM,Derivation) | (G-A437,SRT,"Maximum") | — " — |
| Std_Deviation_s | (126401,DCM,SUVbw) | (121401,DCM,Derivation) | (R-10047,SRT,Standard Deviation) | |
| Volume_s | (G-D705,SRT,Volume) | (G-C036,SRT,Measurement Method) | (126030,DCM,"Sum of segmented voxel volumes") | (ml,UCUM,"Milliliter") |
| RMS_s | (126401,DCM,SUVbw) | (121401,DCM,Derivation) | (C2347976,UMLS,"RMS") | ({SUVbw}g/ml,UCUM,"Standardized Uptake Value body weight") |
| Peak_s | (126401,DCM,SUVbw) | (121401,DCM,Derivation) | (126031,DCM,"Peak Value Within ROI") | ({SUVbw}g/ml,UCUM,"Standardized Uptake Value body weight") |
| Median_s | (126401,DCM,SUVbw) | (121401,DCM,Derivation) | (R-00319,SRT,"Median") | ({SUVbw}g/ml,UCUM,"Standardized Uptake Value body weight") |
| Upper_Adjacent_s | (250139,99PMP,Upper Adjacent Value) | n/a | n/a | (%,UCUM,"Percent") |
| Q1_Distribution_s | (250140,99PMP,Percent Within First Quarter of Intensity Range) | n/a | n/a | (%,UCUM,"Percent") |
| Q2_Distribution_s | (250141,99PMP,Percent Within Second Quarter of Intensity Range) | n/a | n/a | (%,UCUM,"Percent") |
| Q3_Distribution_s | (250142,99PMP,Percent Within Third | n/a | n/a | (%,UCUM,"Percent") |

[1] https://github.com/QIICR/PET-IndiC/blob/master/QuantitativeIndicesCLI/QuantitativeIndicesCLI.cxx

| | | | | |
|---|---|---|---|---|
| | Quarter of Intensity Range) | | | |
| Q4_Distribution_s | (250143,99PMP,Percent Within Fourth Quarter of Intensity Range) | n/a | n/a | (%,UCUM,"Percent") |
| TLG_s [1] | (126033,DCM,Total Lesion Glycolysis) | n/a | n/a | (g,UCUM,"Gram") |
| Glycolysis_Q1_s | (250145,99PMP,Glycolysis Within First Quarter of Intensity Range) | n/a | n/a | (g,UCUM,"Gram") |
| Glycolysis_Q2_s | (250146,99PMP,Glycolysis Within Second Quarter of Intensity Range) | n/a | n/a | (g,UCUM,"Gram") |
| Glycolysis_Q3_s | (250147,99PMP,Glycolysis Within Third Quarter of Intensity Range) | n/a | n/a | (g,UCUM,"Gram") |
| Glycolysis_Q4_s | (250148,99PMP,Glycolysis Within Fourth Quarter of Intensity Range) | n/a | n/a | (g,UCUM,"Gram") |
| SAM_s | (126037,DCM, Standardized Added Metabolic Activity) | n/a | n/a | (g,UCUM,"Gram") |
| SAM_Background_s | (126038,DCM, Standardized Added Metabolic Activity Background) | n/a | n/a | ({SUVbw}g/ml,UCUM,"Standardized Uptake Value body weight") |

## References


1. Larson SM, Erdi Y, Akhurst T, Mazumdar M, Macapinlac HA, Finn RD, et al. Tumor Treatment Response Based on Visual and Quantitative Changes in Global Tumor Glycolysis Using PET-FDG Imaging. The Visual Response Score and the Change in Total Lesion Glycolysis. Clin Positron Imaging. 1999;2: 159–171. Available: http://www.ncbi.nlm.nih.gov/pubmed/14516540
