# Supplementary material for: DICOM for quantitative imaging biomarker development: a standards based approach to sharing clinical data and structured PET/CT analysis results in head and neck cancer research
Source: PeerJ. 2016 May 24;4:e2057. doi: 10.7717/peerj.2057 (PMC4888317; doi:10.7717/peerj.2057)
Supplement: Appendix S4 [file peerj-04-2057-s004.pdf]

Appendix 4: Example of a populated TID 1500 imaging measurements container

Provided below is an example of the structure of the Imaging Measurements and one Measurement Group in one session, with some representative measurements illustrated and others elided. The measurements discussed in this manuscript, encoded as DICOM SR objects and shared in the TCIA QIN-HEADNECK collection, follow the general pattern of this example.

```
CONTAINER: (126010,DCM,"Imaging Measurements")
> CONTAINS: CONTAINER: (125007,DCM,"Measurement Group")
>> HAS OBS CONTEXT: TEXT: (C67447,NCIt,"Activity Session") = "2"
>> HAS OBS CONTEXT: TEXT: (112039,DCM,"Tracking Identifier") = "primary tumor"
>> HAS OBS CONTEXT: UIDREF: (112040,DCM,"Tracking Unique Identifier") = "2.25..."
>> CONTAINS: CODE: (121071,DCM,"Finding") = (M-80003,SRT,"Neoplasm, Primary")
>> HAS OBS CONTEXT: TEXT: (C2348792,UMLS,"Time Point") = "1"
>> CONTAINS: IMAGE: (121191,DCM,"Referenced Segment") = (...,...) [Segment 1]
>> CONTAINS: UIDREF: (121232,DCM,"Source series for image segmentation") = "...,...)
>> CONTAINS: COMPOSITE: (126100,DCM,"Real World Value Map used for measurement") (...)
>> HAS CONCEPT MOD: CODE: (G-C036,SRT,"Measurement Method") = (126410,DCM,"SUV body weight calculation method")
>> HAS CONCEPT MOD: CODE: (G-C0E3,SRT,"Finding Site") = (T-53131,SRT,"base of tongue")

>> CONTAINS: NUM: (126401,DCM,"SUVbw") = 3.58285 ({SUVbw}g/ml,UCUM,"Standardized Uptake Value body weight")
>>> HAS CONCEPT MOD: CODE: (121401,DCM,"Derivation") = (R-00317,SRT,"Mean")

>> CONTAINS: NUM: (126401,DCM,"SUVbw") = 3.17526 ({SUVbw}g/ml,UCUM,"Standardized Uptake Value body weight")
>>> HAS CONCEPT MOD: CODE: (121401,DCM,"Derivation")  = (R-404FB,SRT,"Minimum")

>> CONTAINS: NUM: (126401,DCM,"SUVbw")  = 7.20806 ({SUVbw}g/ml,UCUM,"Standardized Uptake Value body weight")
>>> HAS CONCEPT MOD: CODE: (121401,DCM,"Derivation")  = (G-A437,SRT,"Maximum")

>> CONTAINS: NUM: (126401,DCM,"SUVbw") = 5.90721 ({SUVbw}g/ml,UCUM,"Standardized Uptake Value body weight")
>>> HAS CONCEPT MOD: CODE: (121401,DCM,"Derivation") = (126031,DCM,"Peak Value Within ROI")

>> CONTAINS: NUM: (G-D705,SRT,"Volume") = 3.21039 (ml,UCUM,"Milliliter")
>>> HAS CONCEPT MOD: CODE: (G-C036,SRT,"Measurement Method") = (126030,DCM,"Sum of segmented voxel volumes")

>> CONTAINS: NUM: (126033,DCM,"Total Lesion Glycolysis") = 11.5024 (g,UCUM,"Gram")

...

>> CONTAINS: NUM: (126401,DCM,"SUVbw")= 3.59184 ({SUVbw}g/ml,UCUM,"Standardized Uptake Value body weight")
>>> HAS CONCEPT MOD: CODE: (121401,DCM,"Derivation")  = (C2347976,UMLS,"RMS")
```

```
>> CONTAINS: NUM: (250145,99PMP,"Glycolysis Within First Quarter of Intensity Range") = 4.98963 (g,UCUM,"Gram")

>> CONTAINS: NUM: (126401,DCM,"SUVbw") = 0.253951 ({SUVbw}g/ml,UCUM,"Standardized Uptake Value body weight")
>>> HAS CONCEPT MOD: CODE: (121401,DCM,"Derivation") = (R-10047,SRT,"Standard Deviation")

...

>> CONTAINS: NUM: (250143,99PMP,"Percent Within Fourth Quarter of Intensity Range") = 3.94737 (%,UCUM,"Percent")
>>CONTAINS: NUM: (126037,DCM,"Standardized Added Metabolic Activity") = 3.35859 (g,UCUM,"Gram")
>> CONTAINS: NUM: (126038,DCM,"Standardized Added Metabolic Activity Background") = 2.53669 ({SUVbw}g/ml,UCUM,"Standardized Uptake Value body weight")
```
